# *In vitro–ex vivo* correlations between a cell-laden hydrogel and mucosal tissue for screening composite delivery systems

**DOI:** 10.1080/10717544.2016.1242178

**Published:** 2017-02-21

**Authors:** Anna K. Blakney, Adam B. Little, Yonghou Jiang, Kim A. Woodrow

**Affiliations:** Department of Bioengineering, University of Washington, Seattle, WA, USA

**Keywords:** Nanofibers, hydrogel, explant, antiretroviral, *in vitro*–*ex vivo*

## Abstract

Composite delivery systems where drugs are electrospun in different layers and vary the drug stacking-order are posited to affect bioavailability. We evaluated how the formulation characteristics of both burst- and sustained-release electrospun fibers containing three physicochemically diverse drugs: dapivirine (DPV), maraviroc (MVC) and tenofovir (TFV) affect *in vitro* and *ex vivo* release. We developed a poly(hydroxyethyl methacrylate) (pHEMA) hydrogel release platform for the rapid, inexpensive *in vitro* evaluation of burst- and sustained-release topical or dermal drug delivery systems with varying microarchitecture. We investigated properties of the hydrogel that could recapitulate *ex vivo* release into nonhuman primate vaginal tissue. Using a dimethyl sulfoxide extraction protocol and high-performance liquid chromatography analysis, we achieved >93% recovery from the hydrogels and >88% recovery from tissue explants for all three drugs. We found that DPV loading, but not stacking order (layers of fiber containing a single drug) or microarchitecture (layers with isolated drug compared to all drugs in the same layer) impacted the burst release *in vitro* and *ex vivo*. Our burst-release formulations showed a correlation for DPV accumulation between the hydrogel and tissue (*R*^2^=^ ^0.80), but the correlation was not significant for MVC or TFV. For the sustained-release formulations, the PLGA/PCL content did not affect TFV release *in vitro* or *ex vivo*. Incorporation of cells into the hydrogel matrix improved the correlation between hydrogel and tissue explant release for TFV. We expect that this hydrogel-tissue mimic may be a promising preclinical model to evaluate topical or transdermal drug delivery systems with complex microarchitectures.

## Introduction

Drug-eluting fibers fabricated by electrospinning are a versatile platform for encapsulation and delivery of physicochemically diverse drugs. The ability of this platform to fabricate complex microarchitectures (Okuda et al., 2010; Blakney et al., 2014) as well as control of macroscopic geometry (Ball et al., 2016) is relevant to various prophylaxis and therapeutic applications. For example, Yuan et al. (2015) found that initial release of an anti-inflammatory agent coupled with sustained release of a chemotherapeutic from pH-responsive electrospun fibers resulted in higher life expectancy in a mouse model of hepatocellular carcinoma. Drug combinations are often more challenging to formulate and deliver but necessary to some applications such as cancer treatment (Wong et al., 2006) and HIV prevention (Chen et al., 2015), where the drugs are physicochemically different (e.g. solubility, pKa, etc.). While electrospun fibers allow fabrication of composites of diverse drugs with different release profiles, how drug-specific release kinetics observed *in vitro* are recapitulated *in vivo* for various routes of administration are more challenging to predict. In particular, dosage forms for transdermal or topical delivery where release is unidirectional and not isotropic may exhibit release profiles *in vitro* that are not recapitulated in tissues and cells. For example, Chen et al. (2012) observed much lower *in vivo* release of vancomycin, gentamicin and lidocaine compared to *in vitro* release profiles from poly(lactic-co-glycolic acid) (PLGA)/collagen sandwich-structured nanofibers for topical delivery of wound healing agents.

Conventional methods for measuring drug release or dissolution fail to recapitulate the anisotropic release that is typical of specific *in vivo* administration routes. For example, parenteral and oral delivery routes are suited for vial- or SOTAX-based *in vitro* release experiments because release from the drug delivery system is isotropic. On the other hand, sophisticated and expensive *in vitro* release setups such as Franz cells are needed for evaluation of transdermal and topical dosage forms, which release anisotropically. While Franz cells account for the directionality of different drug delivery systems, they can be costly and the number of available devices limits the number of experiments. Explant tissue from either humans or non-human primates have been used to test safety and pharmacokinetics for a variety of dosages including vaginal delivery of microbicides (Fletcher et al., 2006; Rohan et al., 2010), oral delivery of nanoparticle chemopreventative drugs (Holpuch et al., 2010) and chemoembolization of doxorubicin-eluting beads for treatment of hepatocellular carcinoma (Namur et al., 2011). However, explant tissue is expensive, difficult to obtain, limited in quantity and precludes testing of drug delivery systems over long time periods due to inability to maintain the viability and integrity of tissue in culture.

Poly(2-hydroxyethyl methacrylate) (pHEMA) hydrogels have been widely used in drug delivery and tissue engineering, and are useful for their high water content, porosity, easy fabrication and formulation flexibility. Formulation parameters of pHEMA hydrogels have been well-defined, including altering cross-linking density to control drug diffusion (Hsiue et al., 2001), incorporating specific monomers to enhance drug loading and release properties (Andrade‐Vivero et al., 2007), and varying cross-linker concentration to increase kinetic solubility of amorphous solid dispersions (Sun et al., 2012). Due to the tunability of pHEMA hydrogels, as well as their high water content and inexpensive and rapid fabrication, we hypothesized that these biomaterials could be used as a three-dimensional non-sink testing medium for electrospun fibers with intricate microarchitecture that are designed for transdermal or topical drug delivery applications. A three-dimensional platform more realistically capitulates the *in vivo* environment, while evaluating drug delivery systems in non-sink conditions provides the best discriminating dissolution profiles between formulations (Liu et al., 2013).

In this study, we evaluated how microarchitecture, stacking order and drug loading affect burst-release kinetics and how polymer composition affects sustained-release kinetics. We developed a pHEMA hydrogel *in vitro* release platform for rapid, high-throughput evaluation of electrospun fiber formulations and compared the release profiles between the *in vitro* hydrogel release and the corresponding *ex vivo* mucosal tissue explant release. Finally, we investigated the correlations between release into hydrogels and tissue, and whether incorporation of cells into the hydrogel matrix could improve the *in vivo–ex vivo* prediction of drug concentrations in tissue.

## Materials and methods

### Burst- and sustained-release fiber preparation

Polyvinyl alcohol (M_w_=84–124 kDa, 87–89% hydrolysis) and polycaprolactone (M_n_=80 kDA) purchased from Sigma-Aldrich (St. Louis, MO). PLGA (50:50 DL:PLG, acid terminated, 0.15–0.25 dL/g) was purchased from Lactel (Birmingham, AL). Tenofovir (TFV) and dapivirine (DPV) were generous gifts from CONRAD (Arlington, VA). Maraviroc was purchased from the University of Washington pharmacy (Seattle, WA) and purified in house.

PVA fibers (“burst-release fibers”) were electrospun using a 10% (w/v) solution of PVA in water on a NS 1WS500U (Elmarco Inc., Morrisville, NC) free-surface electrospinning instrument, using the following parameters: 160 mm wire electrode distance, −25 kV collecting electrode, 60 kV spinning electrode and 250 mm cartridge travel distance. Single-drug fibers, used for stacking experiments, were prepared using 20% (wt drug/wt polymer) of DPV, MVC or TFV, or 60% (wt drug/wt polymer) DPV. Equal-loading combination fibers were prepared using 6.67% (wt drug/wt polymer) of DPV, MVC and TFV, for a total drug loading of 20% (wt drug/wt polymer). Higher DPV-loaded fibers were prepared using 6.67% (wt drug/wt polymer) of MVC and TFV, and 20% (wt drug/wt polymer). The drug to polymer ratio was kept constant between the stacked and combined fibers.

PLGA/PCL fibers (“sustained-release fibers”) were electrospun, as previously described (Carson et al., 2015), using a 15% (w/v) solution of PLGA/PCL in 1,1,1,3,3,3-hexafluoro-2-propanol (HFIP) using a needle electrospinning setup. PLGA:PCL content was varied to create four blends: 100:0, 80:20, 80:20 and 0:100, and TFV was added to each polymer solution at 15% (wt drug/wt polymer). The solutions were then extruded from a glass 5 mL syringe with a 22-gauge stainless steel needle at 30–50 μL/min using a NE-1000 syringe pump (Farmingdale, NY), exposed to a voltage of 11.2 kV, and collected on a grounded metal plate at a distance of 10 cm.

All fibers were stored in a vacuum desiccator until further analysis. For scanning electron microscopy (SEM) imaging, fibers were sputter coated for 90 s with Au/Pd and imaged at a magnification of 5000× using a Sirion scanning electron microscope at the University of Washington Nanotechnology User Facility.

### Hydrogel formulation and characterization

Hydroxyethyl methacrylate (HEMA), ethylene glycol dimethacrylate (EGDMA) and benzoin isobutyl ether (BIE) were purchased from Sigma-Aldrich (St. Louis, MO). The pre-polymer solution was prepared with 50% (v/v) deionized water, 49% (v/v) HEMA, 0.05% (v/v) EGDMA and 0.05% BIE. For cell-loaded hydrogels, HeLa cells (NIH AIDS Research Reagent Program, Division of AIDS, NIH, Bethesda, MD) were suspended in PBS at a concentration of 1.514 × 10^5^ cells/mL were used in the pre-polymer solution in the place of water. About 500 μL of pre-polymer solution was added each well of a 48-well plate (11 mm in diameter), and placed under UV light for 10 min. After polymerization, hydrogels were gently removed from the plate, and placed in deionized water at 37 °C. Water was exchanged 3× to rid the hydrogels of any impurities leftover from polymerization. Water content was measured by completely dehydrating hydrogels in a 110 °C oven, measuring the dry weight, then allowing to equilibrate in water at 37 °C, measuring the wet weight, and calculated using the following equation:

$$\%\;\mathrm{Water}=100*\frac{\mathrm{Wet}\;\mathrm{weight}-\mathrm{Dry}\;\mathrm{weight}}{\mathrm{Wet}\;\mathrm{weight}}$$

### Hydrogel extraction validation

Drugs were extracted from the hydrogels by incubating overnight on a rotational shaker at 37 °C. Samples were then vortexed well and filtered through a 0.22 μm PVDF filter (Millipore, Darmstadt, Germany) to remove any remaining debris. Drug concentration was analyzed in triplicate using a Shimadzu Prominence LC20AD UV-HPLC system, with a Phenomenex Luna C18 Column (5 μm, 250 × 4.6 mm) and LC Solutions software. The HPLC methods for DPV, MVC and TFV can be found in Supplementary Table 1.

Hydrogel extraction was validated by polymerizing known amounts of each drug into separate hydrogels, extracting, analyzing using HPLC and calculating % recovery using the following equation:

$$\%\;\mathrm{Recovery}=100\;*\;\frac{\mathrm{Mass}\;\mathrm{drug}\;\mathrm{recovered}}{\mathrm{Initial}\;\mathrm{mass}\;\mathrm{drug}}$$

HPLC methods were validated using a standard curve of drug in dimethyl sulfoxide (DMSO), samples with known amounts of each drug spiked individually and in combination to ensure complete recovery and no peak overlap. Extracted samples were either analyzed within 24 h or store at −20 °C until further analysis.

### *In vitro* hydrogel release

Fibers were cut into circles with a diameter of 9.525 mm. Stacked fibers were prepared by cutting the single-drug fibers individually, orienting the samples in the correct stacking order, and firmly indenting the center of all three layers using forceps in order to keep the layers together. Stacked and combined fibers had approximately the same total mass (∼6 mg) and thickness. At the initiation of a release study, prepared hydrogels were placed in a 48-well plate, 200 μL of PBS was added to the top, and then fiber discs were added to the well. Samples were kept in a 37 °C incubator until the appropriate time-point. At each time-point, the hydrogel was removed from the plate, any remaining fibers were scraped from the top of the hydrogel, and the hydrogel was immediately submerged in 4 mL of DMSO for extraction.

### Tissue extraction validation

Drugs were extracted from tissue by adding 1 mL of DMSO to sample and incubating for 2 h at 37° C to allow DMSO to permeate tissue. After incubation, samples were further homogenized using a Precellys tissue homogenizer (Bertin Technologies, Rockville, MD) at 5000 rpm for 2 × 20 s cycles. Samples were then centrifuged at 5000 rpm for 7 min. Supernatant was removed and filtered using a 0.45 μm filter to remove any remaining debris. Samples were analyzed using identical high-performance liquid chromatography (HPLC) methods as the hydrogel extraction. To validate the methods for the tissue extraction protocol, each batch included a sample of blank DMSO, blank DMSO and tissue eluate, standards prepared in DMSO and tissue eluate, two spiked samples with known amount of drug, and a sample containing all three drugs to confirm that the peaks did not overlap. Retention times and wavelengths for detection were identical to the hydrogel methods for all drugs. Recovery for drugs spiked into untreated tissue samples can be found in Table 1.

**Table 1.** Validation of extraction of DPV, MVC and TFV from hydrogels and tissue explants alone and in the presence of the triple drug formulation.

Drug	% Recovery, hydrogel spike	% Recovery, tissue spike	% Recovery, triple drug spike	Range of quantification (μg/mL)	Limit of detection (μg/mL)
DPV	92.9 ± 16.7	100.4 ± 6.1	99.80	0.1–100	0.01
MVC	108.0 ± 9.75	98.6 ± 4.2	96.40	5–500	0.5
TFV	100 ± 14.5	88.0 ± 3.3	101.00	5–500	0.5

### *Ex vivo* tissue explant release

Whole reproductive tracts of pigtail macaques (*Mecaca nemesetrina*) was purchased from the Washington National Primate Research Center (WaNPRC). Tissue from the vaginal tract only was cut into ∼500 mg sections within 2 h of euthanasia, and placed in a 48-well plate, lumen side up, with 200 μL of DMEM in the bottom of each well. Fiber samples were then placed on top of the tissues, and kept in a 37 °C incubator until the appropriate time-point. At each time-point, any remaining visible fibers were removed from the top of the tissue using forceps, and then each tissue was rinsed in 5 mL of sterile PBS to remove remaining fiber debris. Samples were stored at −80 °C until further processing.

### Statistical analysis

Release data is represented as the average ± standard deviation. For each hydrogel and tissue correlation, a linear regression was carried out between the hydrogel dose (μg drug/g hydrogel/mg fiber) and tissue dose (μg drug/g tissue/mg fiber). A significant correlation was defined as a non-zero slope with *p* < 0.01. Statistical analyzes were done in GraphPad Prism, version 6.0.

## Results

### Burst- and sustained-release fibers exhibit uniform fiber morphology and fiber mat properties

**Figure 1.**
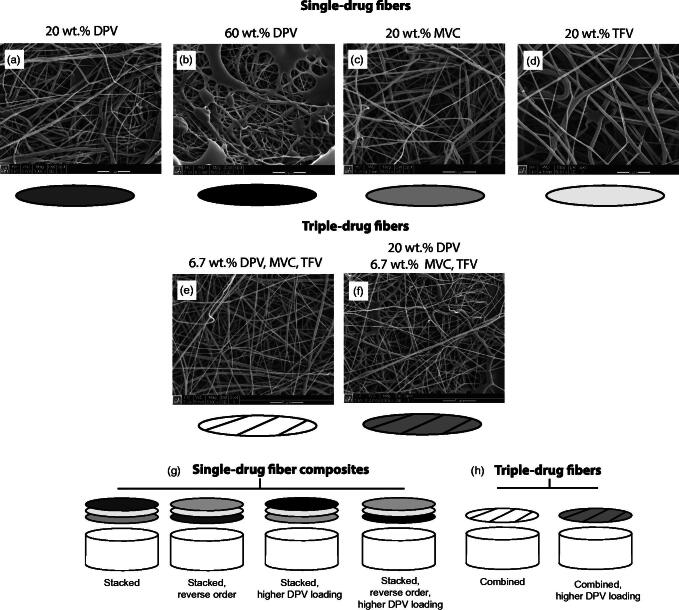
Scanning electron micrographs of electrospun PVA fibers containing either a single-drug (DPV, MVC or TFV) (a–d) or the triple-drug combination (DPV + MVC + TFV) (e-f), and treatment arms of triple-drug combinations (g–h). Scale bars = 5 μm.

**Figure 2.**
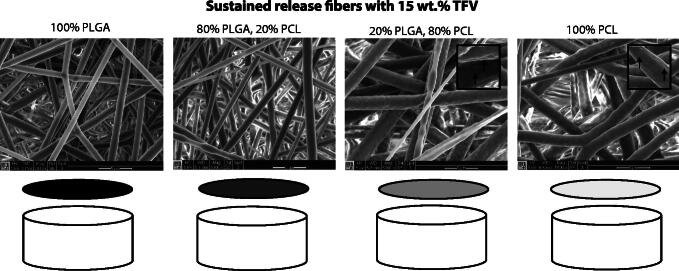
Scanning electron micrographs of electrospun sustained-release fibers containing 15 wt% TFV, and schematic of single-drug sustained-release microarchitecture. Inset shows dimpled fibers in majority PCL samples. Scale bars = 5 μm.

We verified that all fiber formulations exhibited similar fiber density, thickness and fiber diameter since these properties can impact drug release profiles and confound comparisons. SEM showed that PVA fibers containing DPV, MVC and TFV have a round and smooth morphology with a fiber diameter of ∼200 nm (Figure 1). Sustained-release fiber formulations of PLGA/PCL blends containing 15 wt% TFV were also found to have a round and smooth morphology (Figure 2). For the sustained-release formulations, the average fiber diameter of PLGA-dominant fibers was ∼1 μm while PCL-dominant fibers were ∼1.6 μm in diameter. Fibers containing 60 wt% DPV revealed some non-fiber regions (Figure 1b), and PCL-dominant fibers also appeared to exhibit a dimpled a morphology (Figure 2).

DPV, MVC and TFV are physicochemically diverse drugs, and were fabricated as a triple drug combination by either independently isolating them in their own layer or incorporating them together into a single layer. These two variations in formulation allowed us to test whether stacking order and microarchitecture (three drugs in three distinct layers or three drugs in one distinct layer) would impact release kinetics. Single-drug fibers were combined into a composite mat by pressing three layers together to maintain a mat thickness of ∼0.5 mm, which resulted in an equivalent thickness to the fiber formulations containing all three drugs in one layer. Sustained-release fibers showed a consistent thickness of ∼0.7 mm among different blends of PLGA/PCL. Fiber characteristics, including diameter, density and thickness were consistent within burst- and sustained-release fiber groups.

### Validation of hydrogel and tissue extraction methods

We validated a method for efficient extraction of DPV, MVC and TFV from both hydrogel and tissue explant samples to quantify drug concentrations and establish correlations between the *in vitro* and *ex vivo* release profiles. First, we identified conditions that maintained >95% of hydrogel water mass over the course of 72 h as water evaporation over time could confound release kinetics between formulations groups. Hydrogels lost ∼35% water mass due to convective evaporation when placed in a rotational shaker but not when kept in a static incubator with remaining wells filled with water as a source for humidity. Hydrogels and tissue explants were sized to fit exactly into a 48-well plate, with approximate dimensions of 11 mm in diameter and 5 mm in height, and a wet mass of ∼500 mg. For our hydrogels, total drug recovery and extraction efficiency were evaluated using a known amount of each drug spiked directly into the hydrogel precursor solution (Table 1). The extraction protocol involved soaking pHEMA hydrogels or tissue in DMSO for 24 h, and then analyzing an aliquot of the extraction media for drug content using HPLC. For the tissue explants, known amounts of drug were combined with tissue prior to homogenization to ensure that exposure to viable tissue and ceramic beads did not absorb or degrade drugs (Table 1). The extraction procedure was considered valid and acceptable if the drug recovery fell within the range of 100 ± 15%. We measured a hydrogel extraction recovery of 93–108% and a tissue recovery between 88 and 100% for all three drugs. Finally, all three drugs were spiked into a neat sample of DMSO to test whether drug peaks were adequately separated on HPLC for individual detection (Table 1). Each drug showed a separation resolution of at least 0.5 min, including the sample spiked with all three drugs. The triple drug spike resulted in 96–101% recovery for all three drugs. Given these results, our DMSO extraction protocol and HPLC methods are suitable for extraction of DPV, MVC and TFV from both hydrogels and tissue.

### DPV loading, but not stacking order or microarchitecture, enhances burst drug release *in vitro* and *ex vivo*

**Figure 3.**
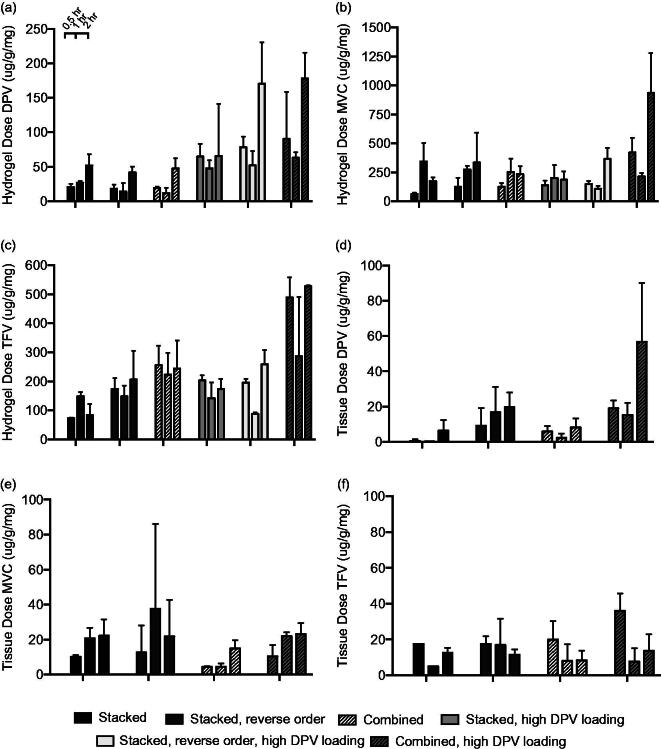
Release profiles of burst fiber formulations of DPV (a and d), MVC (b and e) and TFV (c and f) in hydrogels (a–c) and tissue explants (d–f). Each bar represents the mean of *n* = 3 for hydrogel release and *n* = 4 for tissue explant release, with error bars representing standard deviation.

We compared the effects of stacking order, microarchitecture and DPV drug loading in burst-release PVA fibers on drug release into both hydrogel and tissue. We found that, despite the widely varying physicochemical properties of these drugs, stacking order and microarchitecture (drugs in separate fiber layers versus the same fibers) do not affect DPV, MVC or TFV release into either hydrogel or tissue. For example, the stacked, reverse stacked and combined fibers released a similar amount of DPV in the hydrogel of ∼50 μg/g/mg (Figure 3a) and ∼15 μg/g/mg for the parallel doses in tissue (Figure 3d). However, increasing DPV loading enhanced burst release of DPV, MVC and TFV into hydrogels but only led to enhanced release of DPV into tissue. We found that increasing DPV loading resulted in ∼3-fold higher release rate from the combined fibers after 2 h into both hydrogel and tissue (Figure 3a and d). At 0.5 h in both hydrogel and tissue, DPV also showed a trend of super-saturation as indicated by a sudden increase in drug concentration and subsequent decline. The combined fiber with higher DPV loading showed ∼2-fold higher release of MVC after 2 h in hydrogel but not tissue (Figure 3b and e). Combined fibers with higher DPV loading also had a 2-fold higher release of TFV after 2 h in hydrogel, but not in tissue (Figure 3c and f). Overall, increasing the DPV loading in fibers increased the DPV release rate and total DPV dose delivered after 2 h to both hydrogel and tissue.

**Figure 4.**
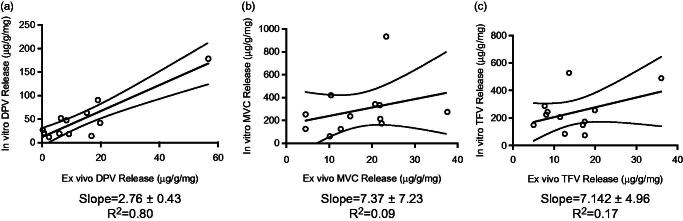
Correlation between hydrogel and tissue explant release profiles for burst-release fiber formulations of (a) DPV, (b) MVC and (c) TFV.

We used linear regression to correlate the dose of drug delivered to the hydrogel and the dose delivered to tissue in order to evaluate whether the *in vitro* hydrogel system could be predictive of *ex vivo* dosing (Figure 4). We found that DPV had a statistically significant correlation (*p* < 0.0001) and non-zero slope, while the correlations for MVC and TFV were not statistically significant (*p* = 0.33 and 0.18, respectively). These results indicate that this pHEMA formulation may be useful for assessment of DPV burst release, but not MVC or TFV.

### PLGA/PCL content does not alter release rate of TFV *in vitro* or *ex vivo* but incorporating cells into hydrogel improves hydrogel/tissue release correlation

**Figure 5.**
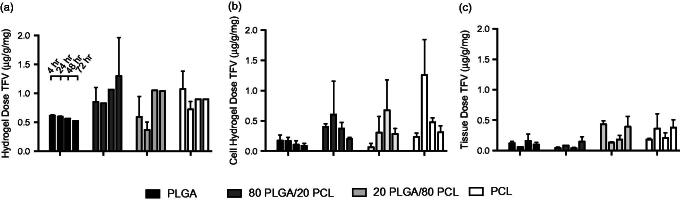
Release profiles of sustained-release fiber formulations loaded with 15 wt% TFV in (a) hydrogel, (b) hydrogels with encapsulated cells and (c) tissue explants. Each point represent *n* = 3 for hydrogel samples and *n* = 4 for tissue samples, with error bars representing standard deviation.

In order to evaluate the hydrogels for informing release from sustained formulations and to compare with previously observed sink *in vitro* release profiles (Carson et al., 2015), we assessed how the ratio of PLGA to PCL affects release of TFV into hydrogels and tissue. We observed a slight trend for PLGA-dominant fibers to enhance sustained release of TFV compared to PCL-dominant fibers (Figure 5). For example, ∼0.5 μg/g/mg TFV was released into the hydrogel for the PLGA-only formulation after 72 h compared to ∼1.0 μg/g/mg for the PCL-only formulation (Figure 5a).

**Figure 6.**
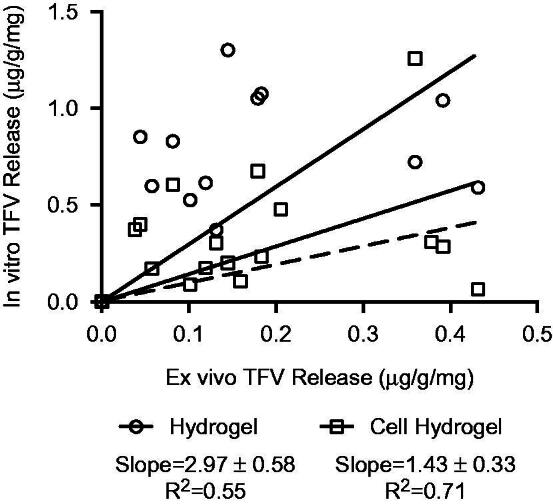
Correlation between hydrogel and tissue explant release profiles for burst-release fiber formulations.

We hypothesized that encapsulating mammalian cervical epithelial cells into pHEMA hydrogels could improve the bulk composition of the hydrogel matrix to have more tissue-like properties and improve the correlation between drug release with tissue. We entrapped approximately 75 000 HeLa cells into each hydrogel and since these hydrogels undergo UV polymerization, it is unlikely that any of the cells remained viable post-polymerization. However, cell viability is unimportant for our purpose since our aim was only to impart physicochemical properties of cells into the bulk hydrogel matrix rather than recreating a metabolically active tissue-mimic. We measured TFV released into cell-laden hydrogels compared to release into hydrogels-only and tissue explants (Figure 5). After 72 h, TFV release was ∼1.0 μg into hydrogels, ∼0.5 μg into cell-laden hydrogels, and ∼0.4 μg into tissue explants. There were no significant differences between any of the sustained-release formulations in hydrogel-only, cell-laden hydrogel, or tissue. We next evaluated the correlation between released TFV content into the hydrogel compared to tissue, and also between cell-laden hydrogel and tissue (Figure 6). A slope of unity would predict the same dosage being released into both the hydrogel and tissue. Linear regression between drug content measured in hydrogel-only and tissue had a measured slope of 2.20 ± 0.53 (*R*^2^=0.55), indicating that ∼2.2 mg TFV are released into the hydrogel for every 1 mg released into tissue. In contrast, the drug content correlation between tissue and cell-laden hydrogel had a slope of 1.43 ± 0.33 (*R*^2^ of 0.71), indicating that ∼1.4 mg are released into cell-hydrogel for every 1 mg released into tissue. Thus, incorporation of cells improved fit and linearity of the correlation between hydrogel and tissue release of TFV.

## Discussion

We investigated hydrogels as a non-sink release platform that could discern differences in release from fiber meshes with varying microarchitecture and drug loading. We found that pHEMA hydrogels could recapitulate *ex vivo* release of DPV from burst-release formulations and release of TFV from sustained-release formulations. However, the correlations between hydrogel and tissue release were not significant for burst-release formulations of MVC and TFV. In addition, incorporating cells into pHEMA hydrogels improved the correlation between TFV release in hydrogel and tissue.

For our burst-release formulations, we found that there was a significant correlation between DPV release into hydrogel and tissue, which is likely due to the greater solubility of DPV in the hydrogel matrix compared to either MVC or TFV, which are more water-soluble. The large error observed for some of the fiber formulations is likely a result of inherent variation in the hydrogels, tissue, and extraction efficiency. We expect that minimizing variation in these experimental conditions could improve hydrogel-tissue correlations for MVC and TFV. One strategy to formulate the hydrogel to improve correlation for MVC and TFV release is to lower the cross-linking density, which has previously been shown to accelerate release kinetics of a water soluble drug (pilocarpine) out of the hydrogel matrix (Hsiue et al., 2001). Alternatively, a more hydrophilic, longer cross-linking group, such as tetraethylene glycol diacrylate (TEGDA) or polyethylene glycol diacrylate (PEGDA) could be used to create a more porous, hydrophilic hydrogel that could also increase diffusion of hydrophilic drugs (Mabilleau et al., 2006). We expect that pHEMA hydrogels may also be used to evaluate DPV release from other solid dosage forms that are placed proximal to tissue and release profiles can be manipulated by the formulation, such as films or rings (Romano et al., 2009; Devlin et al., 2013).

Overall, we observed that DPV and TFV show a trend towards a higher amount of released drug at the 0.5 h time-point compared to the 1 h time-point. A release profile wherein the drug amount peaks and then sharply declines is a characteristic of supersaturation (Sun & Lee, 2015b), and suggests that the drug reaches a critical saturation point and then crashes out of solution. The observed trends for DPV and TFV could be due to supersaturation of drugs in the hydrogel, which has previously been observed to occur from medium-soluble carrier films in non-sink conditions (Sun & Lee, 2015b). PVA is water-soluble but likely less soluble in pHEMA hydrogel and tissue, which would lead to a dissolution-controlled mechanism and then potential to supersaturate both the hydrogel and tissue with amorphously dispersed drugs. TFV and MVC were previously shown to create amorphous solid dispersions in electrospun fibers (Ball & Woodrow, 2014; Blakney et al., 2014), and while we did not test the crystallinity of these drugs in our fibers, we expect that these drugs rapidly dissolve from PVA fibers into the hydrogel matrix. This outcome would results in initial super-saturation but eventually nucleation and crystallization, leading to a decrease in concentration at the 1 h time-point (Sun & Lee, 2015a).

Carson et al. (2015) previously observed that increasing PLGA content in PLGA/PCL blend fibers sustains TFV release *in vitro* under sink conditions. We also observed that increasing PLGA content showed a trend for sustaining TFV release in both hydrogels and explant tissue, but the sustained-release phase was more modest. It is possible that the hydrogel and explant release systems are not solely dissolution controlled as with sink conditions for *in vitro* release, and thus drug release profiles in the two conditions would be inherently different. Experimental error due to variation in hydrogel and tissue samples and extraction efficiency could also mask trends in sustained TFV release. With an *in vitro* hydrogel and *ex vivo* tissue release system, the fibers are not completely surrounded by fluid and there is only minimal fluid convection, so the release kinetics may be limited by wetting of the fibers and the dissolution of TFV. Thus, the previously observed release profiles of TFV from PLGA/PCL blends in sink conditions *in vitro* may be entirely different in non-sink conditions, as expected. In parallel, we had previously observed that fiber microarchitecture does impact hydrophilic drug release *in vitro*, which was not observed for the triple-drug combination fibers in these experiments (Blakney et al., 2014).

We incorporated HeLa cells into our pHEMA hydrogels to assess whether imparting a cellular makeup to the hydrogel matrix could improve the correlation between the hydrogel and tissue sustained TFV release kinetics. Although live cells have been incorporated into hydrogels (Hwang et al., 2010; Fu et al., 2012), our aim was only to recapitulate the overall bulk material properties of tissue using a simple hydrogel system. In order to avoid defining and attempting to formulate the exact composition of tissue, such as the percentage proteins, lipids, sugars, etc., the cell itself presented the most obvious reagent. While a cell-laden hydrogel is similar to tissue on a bulk matrix level, one downfall is that this system cannot recapitulate metabolism since we did not attempt to maintain cell viabilty. Thus, hydrogels and cell-laden hydrogels differ strictly in the composition of the bulk hydrogel matrix, and it is likely that TFV solubility in the cell-laden hydrogel more closely matches the solubility of TFV in the tissue matrix. It is possible that by further increasing the number of cells in each hydrogel, the correlation between the dosage of drug into the hydrogel and tissue could be improved. Given the broad formulation potential of pHEMA hydrogels, we expect that this *in vitro* release platform could be extended to other topical routes such as oral, nasal or dermal delivery.

## Conclusion

We evaluated how electrospun fiber formulation affects the *in vitro* and *ex vivo* release profiles for three physicochemically diverse drugs. A novel cell-laden hydrogel platform was developed for the rapid, inexpensive *in vitro* evaluation of burst- and sustained-release electrospun fiber formulations of varying microarchitecture. The hydrogel-tissue mimic was used to establish *in vitro–ex vivo* correlations with release into vaginal mucosal tissue explants. To establish the correlations, we first validated the extraction and analysis using HPLC from both the hydrogel and tissue for DPV, MVC and TFV. We show that the stacking order and microarchitecture did not affect burst-release kinetics, whereas increasing DPV loading enhanced release of all three drugs from the composites. For our rapid-release PVA formulations, drug release into the hydrogel correlated to release into tissue for DPV but not MVC or TFV. For our sustained-release polyester formulations, the ratio of PLGA to PCL did not affect the sustained release of TFV in either the hydrogel or tissue. Incorporating a model cell type into the hydrogel improved the correlation between the *in vitro* and *ex vivo* release of sustained TFV formulations. Overall, the versatility of the pHEMA hydrogel system may offer a new approach for rapid evaluation and optimization of topical and transdermal drug delivery dosages *in vitro* to predict *in vivo* performance.

## Supplementary Material

Supplementary_Table_1.docx

## Notes

**Supplementary material available online.**
