# Supplementary material for: In vitro–ex vivo correlations between a cell-laden hydrogel and mucosal tissue for screening composite delivery systems
Source: Drug Deliv. 2017 Feb 21;24(1):582–90. doi: 10.1080/10717544.2016.1242178 (PMC5594105; doi:10.1080/10717544.2016.1242178)
Supplement: Supplementary_Table_1.docx [file IDRD_A_1242178_SM8816.docx]

| **Supplementary Table 1.** HPLC method specifications for the detection of DPV, MVC and TFV from extracted hydrogel and tissue samples. | | | | | | | |
| --- | --- | --- | --- | --- | --- | --- | --- |
| **Drug** | **Mobile phase** | **Flow rate (mL/min)** | **Method time (min)** | **Column oven temperature (°C)** | **Injection volume (μL)** | **Detection wavelength (nm)** | **Retention time (min)** |
| DPV | 65% ACN + 35% 10 mM ammonium acetate in H_2_O | 1.0 | 10 | 30 | 10 | 310 | 6.4 |
| MVC | 26% ACN + 74% 50 mM KH_2_PO_4_ in H_2_O, pH=3.2 | 1.0 | 15 | 44 | 20 | 193 | 8.9 |
| TFV | 28% ACN + 72% 0.045% TFA in H_2_O | 1.0 | 10 | 30 | 20 | 259 | 2.3 |
